# Family-Based Treatment for Anorexia Nervosa Symptoms in High-Risk Youth: A Partially-Randomized Preference-Design Study

**DOI:** 10.3389/fpsyt.2019.00985

**Published:** 2020-01-22

**Authors:** Katharine L. Loeb, Ruth Striegel Weissman, Sue Marcus, Cassandra Pattanayak, Lisa Hail, Kelly C. Kung, Diana Schron, Nancy Zucker, Daniel Le Grange, James Lock, Jeffrey H. Newcorn, C. Barr Taylor, B. Timothy Walsh

**Affiliations:** ^1^ School of Psychology, Fairleigh Dickinson University, Teaneck, NJ, United States; ^2^ Department of Psychiatry, Icahn School of Medicine at Mount Sinai, New York, NY, United States; ^3^ Department of Psychology, Wesleyan University, Middletown, CT, United States; ^4^ Consultant, Philadelphia, PA, United States; ^5^ Department of Mathematics, Quantitative Reasoning Program, and the Quantitative Analysis Institute at Wellesley College, Wellesley, CT, United States; ^6^ Department of Psychiatry, University of California, San Francisco, San Francisco, CA, United States; ^7^ Department of Mathematics, Boston University, Boston, MA, United States; ^8^ School of Dentistry, University of California, San Francisco, San Francisco, CA, United States; ^9^ Department of Psychiatry and Behavioral Sciences, Duke University School of Medicine, Durham, NC, United States; ^10^ Department of Psychiatry and Behavioral Neuroscience, The University of Chicago, Chicago, IL, United States (Emeritus); ^11^ Department of Psychiatry and Behavioral Sciences, Stanford University School of Medicine, Stanford, CA, United States; ^12^ Departments of Psychiatry and Pediatrics, Icahn School of Medicine at Mount Sinai, New York, NY, United States; ^13^ Center for m^2^Health, Palo Alto University, Palo Alto, CA, United States; ^14^ Department of Psychiatry, Columbia University, New York, NY, United States

**Keywords:** anorexia nervosa, early identification, early intervention, family-based treatment, partially-randomized preference design

## Abstract

This pilot study adapted family-based treatment (FBT) for youth with potentially prodromal anorexia nervosa (AN). Fifty-nine youth with clinically significant AN symptom constellations, but who never met full Diagnostic and Statistical Manual of Mental Disorders (4th ed., text rev.) (DSM-IV) criteria for AN, were enrolled in a partially randomized preference design study. Participants were offered randomization to FBT or supportive psychotherapy (SPT); those who declined to be randomized because of a strong treatment preference were entered into a parallel, non-randomized self-selected intervention study. Without accessing outcome data, an observational analysis with three diagnostic subclasses was designed based on AN symptom severity profiles, combining randomized and non-randomized participants, such that participants receiving FBT and SPT within each subclass were similar on key baseline characteristics. Outcomes of this pilot study were explored by calculating effect sizes for end-of-treatment values within each subclass, and also with a longitudinal mixed effect model that accounted for subclass. Weight trajectory was measured by percent expected body weight. Psychological outcomes were fear of weight gain, feeling fat, importance of weight, and importance of shape. Results show that the pattern of symptom observations over time was dependent on subclass of SAN (least symptomatic, moderately symptomatic, or most symptomatic) and on the target outcome variable category (weight or psychological). Results from this study, which should be considered in the context of the small sample sizes overall and within groups, can generate hypotheses for future, larger research trials on early treatment strategies. Feasibility findings illustrate how the innovative partially randomized preference design has potential broader application for AN intervention research.

**Clinical Trial Registration:**

ClinicalTrials.gov, identifier NCT00418977.

## Introduction

Anorexia nervosa (AN) typically onsets in adolescence, with medical and psychiatric sequelae often appearing even before the diagnostic threshold is crossed (1, 2). The compromised weight status inherent in a diagnosis of AN, whether achieved by weight loss or, for some younger individuals, failure to gain weight along an expected growth curve trajectory, is a process that occurs by degrees. As such, the AN syndrome is invariably preceded by a prodromal state (3–5), although during the prospective symptom development phase, there are no definitive markers of risk for full AN (4). Specifically, it is not known with precision for which adolescents the AN features will progress, will remain at a chronically sub-diagnostic level, or will be transient. Research has shown that all three outcomes are possible, although conclusions from longitudinal epidemiological studies of AN are limited by the low prevalence of the disorder (6–10).

The consensus in the field is that early identification and treatment of the emerging signs and symptoms of AN are critical in mitigating the risks associated with this pernicious eating disorder and in conferring an improved prognosis (11–15). That said, targeted early intervention efforts for AN are predicated on an accurate assessment of its diagnostic criteria, a task dually challenged by developmental factors as well as illness-specific ones (16). For example, expected body weight (EBW) among children and adolescents is an individualized, moving target; in turn, determining degree of deviation from this reference point requires both studying historical healthy growth curves, and modeling future ones that account for growth in age and stature over time (17). In addition, patient report of phenomena such as fear of weight gain and undue influence of shape and weight on self-concept relies on abstract cognitive capacities that are still under development in youth (18–20). Moreover, the ego-syntonic nature of AN frequently precludes direct admission of such symptoms (21). Thus, one complication in the early identification and treatment of potentially prodromal AN is missed “caseness” of frank AN. The diagnostic revisions to AN in the 5th Edition of the *Diagnostic and Statistical Manual of Mental Disorders* (DSM-5; 22) are designed, in part, to render the criteria more developmentally sensitive (9, 18, 19, 23, 24).

On the continuum of eating disorder intervention, early treatment falls at the cusp between targeted prevention of high risk individuals, and focused treatment of those already diagnosed (25). Given that a prodromal state is only accurately labeled as such in retrospect (i.e., after a full disorder develops), and targeting a potential psychopathological prodrome carries ethical risks such as unnecessary treatment and stigmatization among false positives (26), careful consideration is warranted before intervening. In the case of incipient AN, both research and expert consensus support taking action (19). The literature has long since characterized sub-diagnostic cases and argued that they are not markedly distinct from their full-AN counterparts in medical complications, comorbidities, and outcomes (2, 27–31). While these sub-diagnostic samples are generally heterogeneous with regard to course, they collectively include a portion of individuals who have not yet met the diagnosis and are at risk for progression to AN. Moreover, not only is the sub-syndromal AN symptom profile clinically significant in its own right (thus often meeting criteria for the residual diagnostic category of Eating Disorder Not Otherwise Specified or the later Otherwise Specified Feeding or Eating Disorder), but the impending AN syndrome is arguably severe enough to warrant risking treating cases ultimately revealed to be false positives.

What is yet unanswered in the literature is the optimal form of early intervention, to both resolve extant symptoms and prevent conversion to the full AN syndrome. This is particularly important to address in the stages of development when risk for AN onset is the highest.

While one community-based prevention study tested a brief, parent-based internet intervention for high risk girls (32), no randomized controlled trials (RCT) to date have focused on treatment-seeking youth who are potentially prodromal by virtue of a clinically significant, AN-like symptom constellation.

The current pilot study aimed to assess the feasibility and effect estimates for two interventions for children and adolescents with sub-syndromal DSM-IV (4th ed., text rev.; *DSM–IV–TR*; 33) AN (SAN), to inform the design and hypotheses of future, larger research trials (34) on early treatment strategies. Specifically, we evaluated family-based treatment (FBT) and individual supportive psychotherapy (SPT) for individuals exhibiting proximal risk for conversion to AN based on their symptom profile, while never having met full criteria for AN. In light of the concerns regarding missed “caseness” outlined above, we included youth with both stringent and relaxed study-specific criteria for SAN (see *Method*). Based on the literature supporting the efficacy of FBT for AN (35), we anticipated that effect estimates would favor an adaptation for high-risk youth, relative to a control intervention in (a) reducing the severity of the emerging or present AN diagnostic symptoms (specifically low weight status, fear of weight gain, disturbance in the experience of shape and weight, and overvaluation of shape and weight), and (b) decreasing the likelihood of developing full AN during the observation period.

## Method

### Study Design Development

This pilot study, part of a National Institute of Mental Health Career Developmental Award (K23 MH074506-01) granted to the first author, was originally intended to employ a pure RCT design. However, during the development and early phases, a strong and increasing preference for FBT among carers of potential participants became evident, as manifested by a steadily rising rate of declining to risk randomization assignment to a study intervention other than FBT. Specifically, within a 16-month period, among potential participants initiating telephone inquiries for the study and declining based on a stated reason, 0% cited this concern in the first quarter, 50% did so in the second quarter, 67% in the third, and 75% in the fourth.

These data posed two important issues: a practical one regarding feasibility of recruitment and a conceptual one regarding the importance of treatment preference as a variable to study in its own right. We thus implemented a simultaneous parallel, non-randomized trial in which families who declined randomization were offered their intervention of choice and followed with the identical study protocol for assessment and treatment as their randomized counterparts. Importantly, this partially randomized preference design avoids non-consent bias, i.e., restricting the observed sample to only those willing to risk randomization to an intervention other than FBT, in light of clear population-level variability on this characteristic. Non-consent may correlate with treatment response, or represent a proxy for another variable associated with differential response to FBT, thereby introducing sample bias if not addressed methodologically. Additionally, a partially randomized preference study permits objective, quantitative methods for potentially combining randomized and non-randomized groups for analysis (36–38).

### Ethics Approval

The dual research protocols (randomized and non-randomized) were approved by the Icahn School of Medicine at Mount Sinai's Institutional Review Board.

### Participants

This pilot study aimed to enroll 60 participants across intervention conditions. Participants were male and female children and adolescents, ages 9–18 years, who met criteria for SAN as defined below, were living with parent(s) or guardian(s) willing to participate in the study intervention and able to provide consent in English, and were deemed medically stable for outpatient treatment by their physician. For the non-randomized study, declining randomization was an additional inclusion criterion. Exclusion criteria were: a history of full AN; current psychosis, substance use disorder, or active suicidality; current antipsychotic medication; medical or physical conditions known to influence eating, weight, or menstrual status; refusal to agree to engage in ongoing medical management with a physician or permit ongoing exchange of clinical information with the treating physician; refusal to agree for the research team to obtain weight and height at study sessions; and current or previous participation in FBT. Other concurrent treatment, including psychological or psychopharmacological interventions, was permitted to increase generalizability to clinical populations, and randomization was stratified for this variable. For participants with a history of sexual or physical abuse by parents, siblings, or guardians, perpetrators of the abuse were excluded from participation in the study intervention.

As noted above, SAN was operationalized broadly to intentionally capture a heterogeneous sample of at-risk youth. Specifically, the purpose of including a “relaxed” SAN criteria profile (i.e., lenient by virtue of lower clinical thresholds as well as interpretation of symptom indicators), in addition to the more stringent one, was to target a similar profile of individuals who may be at risk of progressing to AN without intervention, by virtue of exhibiting clinically significant restrictive dietary habits leading to concerning weight loss. **Table 1** presents the study criteria for SAN.

**Table 1:** Study Criteria for SAN.

Features	Definition of SAN	
	Strict	Lenient
**Number of DSM-IV AN criteria (A** ^1^ **, B** ^2^ **, C** ^3^ **, D** ^4^ **) currently met** **History of full-criteria AN** **Adjustment for males**	2-3 No Criterion D imputed as present if Criterion A met	2-3 No Criterion D imputed as present if Criterion A met
**Adjustment for pre-menarcheal** ^5^ **females** **Adjustment to Criterion A** ^5,6^ **Adjustment to Criterion B** ^7^	Criterion D imputed as present if Criterion A met Not allowed Not allowed	Criterion D imputed as present if Criterion A met Allowed Allowed

SAN, sub-syndromal DSM-IV anorexia nervosa. AN, anorexia nervosa.

^1^Criterion A = refusal to maintain normal body weight, strictly interpreted as at or above 85% of expected body weight by population norms.

^2^Criterion B = fear of weight gain.

^3^Criterion C = disturbance in experience or valuation of body weight/shape.

^4^Criterion D = amenorrhea.

^5^Primary amenorrhea was defined in this study as delayed menarche beyond age 16.

^6^If the strictest interpretation of Criterion A was not met, participants must have engaged in dietary restriction leading to a below-expected weight by population norms or individualized growth curve history, in combination with 2–3 of the remaining criteria for inclusion in the study.

^7^For cases with adjusted Criterion A only, extreme self-directed dietary restriction that interfered with weight gain efforts was regarded as behavioral evidence of Criterion B even if such fear was not verbally endorsed. This determination was informed by clinical assessment of both recent typical eating patterns and the participant's reaction to any attempts at renourishment, as reported by patient, parent, and/or referring clinician.

### Recruitment

Participants for this study were recruited by informing colleagues, pediatricians, organizations, and other clinics treating eating disorders of the protocol. No recruitment efforts revealed the availability of the non-randomized preference arm in order to maintain the integrity of the randomized study and to ensure that those entering the parallel study were truly declining randomization. Referral sources (e.g., pediatricians) who became aware of the parallel arm were briefed on the purpose and importance of describing only the primary study to potential participants, and agreed to abide by the research protocol.

### Baseline Assessment

Potential participants were screened for preliminary study fit in a brief telephone screening interview, followed by an in-person assessment session to obtain informed consent and assent and evaluate eligibility. Inclusionary and exclusionary criteria were assessed with clinical interviews with the patient and parent(s) that included questions derived from the Schedule for Affective Disorders and Schizophrenia for School-Age Children**—**Present and Lifetime Version (K-SADS; 39) and the Eating Disorder Examination, edition 16.0D (EDE; 40) to rule out a history of full AN. The EDE was also used to rule in current SAN.

### Randomization

Randomization to study intervention was programmed by author BTW—who was uninvolved in any other study operations—using computer-generated assignment. Random allocation assignments were stored in envelopes sequentially numbered, filled and sealed by a research assistant external to the study team. Research assistants within the study team enrolled participants. For those parents or patients declining randomization at any point during the assessment process, the parallel non-randomized study was offered, and allocation to FBT or SPT was determined by preference following an alternate consent/assent procedure. All other study procedures remained identical for the randomized and non-randomized arms of the study.

### Intervention and Medical Management Settings

The study intervention setting was a specialist eating disorders program within an academic medical center. Initial medical clearance and ongoing medical oversight, obtained external to the study and research setting, were required for all participants in the study. These were provided by participants' established pediatricians or by a specialist referral, if requested by the family, within or outside of the hospital system. Medical management visits with the physician were required to take place at least monthly, or more frequently as dictated by the physician's judgment of the patient's clinical severity. Physicians treating the participants in this study were provided with the Society for Adolescent Health and Medicine's [(41); since updated as (17)] guidelines for medical management and criteria for hospitalization with youth with restrictive eating disorders.

### Interim and Outcome Assessment

Height and weight were obtained on a physician's scale as part of the EDE and at every visit for the purpose of tracking weight change and growth in participants. Percent EBW at each session, a primary outcome variable, was derived from these data by calculating current weight as a percentage of weight corresponding to 50^th^ percentile body mass index (weight in kg/height in m^2^) percentile adjusted for age and sex (42). Participants were weighed in single-layer street clothes, without shoes or heavy accessories.

If a participant's clinical status worsened to the extent that s/he was no longer medically or psychiatrically stable for outpatient treatment or developed full AN, the case was regarded as a study intervention failure and the participant was exited from the study with a referral to a more intensive level of care. Study attrition (investigator- or participant-initiated exit from the research) cases were not replaced.

The primary outcomes were changes in severity of key diagnostic dimensions (weight status, fear of weight gain, disturbance in the experience of shape and weight, and overvaluation of shape and weight) and diagnostic status (conversion to AN). The study included four major assessment points (before treatment, end-of-treatment or termination/drop-out, and 6 months and 12 months post-treatment), as well as checks for changes in diagnostic indices at every session. The current paper focuses on the study's primary hypotheses and corresponding outcome variables, within the active, 14-session intervention observation period.

### Baseline, Outcome, and Classification Measures

#### Eating Disorder Examination (EDE)

The EDE edition 16.0D (40) interview generates eating disorder diagnoses, four severity subscales, a global score, and frequencies of key eating disorder behaviors. The EDE was originally developed for adults but has also been used successfully with adolescents (43). It has sound psychometric properties (44) and is sensitive to change. The diagnostic items of EDE, including fear of weight gain, feeling fat, importance of weight, and importance of shape, correspond to DSM-IV criteria, and were used in this study to determine baseline eligibility (using the 3-month EDE timeframe for diagnosis), in the generation of the subclass profiles described below (three-month timeframe), and in the evaluation of treatment outcome (1-month timeframe, per research convention, to avoid significant overlap with course of treatment). Denial of seriousness of low body weight per DSM-IV criteria is not directly captured in the EDE, and therefore an item assessing this was added to the EDE for purposes of diagnosis, subclass analyses, and tracking potential conversion to AN, which would necessitate withdrawing the participant from the research.

#### Eating Disorders Examination—Parent Version (PEDE)

The PEDE (16, 45), adapted from the EDE, was developed to systematically assess for the parent's perspective on the patient's eating disorder features, and it includes specific queries for behavioral indicators that a symptom may be present (46). It was administered at the same intervals as the EDE. The diagnostic items from the PEDE were used in conjunction with the EDE to create the subclass profiles as described below. The PEDE's construct validity and internal consistency are established as on par with the psychometric properties of the EDE (46).

#### Eating Disorders Examination Questionnaire (EDEQ)

The EDEQ is a questionnaire version of the EDE (47); it is reliable and valid (44), and regularly used in adolescent samples (43, 48, 49). Like in the EDE, an item was added to the EDEQ to evaluate denial of seriousness of low weight. In addition to its use at the major assessment time points, the EDEQ was administered in a modified form at each session to assess the period of time elapsed since the prior session. This session-based questionnaire, along with height and weight measurements at each session, enabled monitoring to determine if any participant's diagnostic status had converted to full AN, e.g., by virtue of further weight loss or endorsement of additional cognitive symptoms, at any point during the intervention phase of the study. In instances when end-of-treatment data were not available *via* the EDE interview, scoring from the EDEQ was substituted to minimize missing data.

### Study Interventions

#### Intervention Delivery

Each study intervention was manual-based and consisted of 14 50-minute sessions over 6 months (weekly x eight sessions, biweekly x four sessions, monthly x two sessions). While the standard version of FBT for AN is a 20-session protocol, a briefer treatment course has demonstrated success (e.g., 50) and was deemed a suitable foundation for a sub-syndromal sample. The manuals were initially tested with a small series of pilot participants. Study therapists were clinical psychologists or advanced doctoral psychology trainees; therapists were trained with didactics, role-play, and active rehearsal of both interventions. The principal investigator (PI; KL) conducted weekly group supervision meetings that included review of videotaped sessions. A senior study consultant (DLG) reviewed a randomly selected subset of 20% of sessions across both modalities for adherence assessment and qualitative expert feedback. If problems in the application of the study interventions were identified during these reviews, supervision to correct them was conducted by DLG in conjunction with the PI.

#### Family-Based Treatment (FBT)

FBT for AN (51) is a symptom-focused intervention designed to enlist parents as the agents of their child's recovery, titrating down their involvement as the eating disorder recedes. FBT progresses across three phases: phase one targets renourishment, interruption of behavioral symptoms, and role structuring of family members (parents, patient, siblings); phase two fosters gradual restoration of independence around energy intake and expenditure; and phase three addresses normal adolescent development, relapse prevention, and termination. Each FBT session begins with a brief individual meeting with the patient to obtain weight and discuss any of the adolescent's concerns; the remainder of the session is conducted with the family present. FBT was adapted for SAN (for a full description of the adaptation, see 52–54). This adaptation emphasizes the risk for conversion to full AN in addition to the medical, psychiatric, and functional impairment posed by extant eating disorder symptoms, and prescribes strategies that draw from the broader eating disorders risk and prevention literatures, including family meals and parental modeling of healthy, flexible, non-restrictive eating.

#### Supportive Psychotherapy (SPT)

SPT is a short-term, non-directive individual therapy that represents a credible control intervention by incorporating non-specific therapeutic strategies without including any putative active therapeutic techniques for eating disorders, such as direct prescriptions for healthy eating or the modification of distorted body image experience. SPT was originally developed for intervention trials of bulimia nervosa in adults (55, 56) and was later adapted for use in adolescent research (57). In the current study, parents were included for brief check-ins, in reverse parallel to the proportion of time spent in individual contact with the patient in FBT. Parents were also provided with a basic psychoeducation handout on AN, including the high number of calories typically required for weight restoration. No direct intervention instructions were given to parents. Like FBT, SPT is delivered in three phases, with the first focused on establishing a strong therapeutic alliance, obtaining a complete picture of the eating disorder and its development, and identifying underlying problems that might contribute to the current symptoms. The second phase encourages further exploration and explication of emotional states and difficulties, and the third addresses how residual psychological challenges my affect future adjustment, highlights progress, and processes termination.

### Statistical Analyses

#### Subclass Generation

Following Rubin (58, 59), we designed the study to parallel the ideal, hypothetical randomized experiment that would have been conducted had we been able to randomize all participants. Specifically, without access to outcome data, we created three subclasses of participants such that the individuals receiving FBT and the individuals receiving SPT within each subclass were as similar as possible on baseline characteristics. These subclasses parallel the strata in a hypothetical stratified randomized experiment, and we analyzed the outcomes by comparing FBT and SPT participants within each subclass, to account for baseline characteristics. Both participants who consented to randomization and those who declined are included in the subclasses. We pooled the randomized and non-randomized participants for analysis because the sample size in each of these groups was too small to justify separate analyses. In addition, because there were only 22 randomized participants, the FBT and SPT participants in the randomization group differed from each other on their baseline AN criteria, such that including the randomized participants in the subclassified design improved balance between FBT and SPT on baseline AN criteria. This approach, which explicitly prioritizes balance on the highest-priority covariates, is similar to post-stratification in a purely randomized study, where chance imbalances on key covariates are addressed by creating subclasses as if the randomization had not taken place (60–63).

We first sorted the available baseline variables by investigator consensus regarding clinical priority. Baseline AN criteria were given highest priority, along with EDE Global Score as a broader indicator of eating disorder pathology. **Table 2** shows the highest- and high-priority baseline variables, by subclass and treatment group^1^. We then created a study design by iterating between proposing ways to subclassify the participants, comparing FBT and SPT participants within the proposed subclasses on baseline characteristics, and refining the proposed subclasses to create better balance on these characteristics. Because outcomes were not used during subclass creation, this iterative study design process did not introduce bias.

**Table 2:** Higher-Priority Baseline Clinical Characteristics for Establishing Balanced Treatment Groups, by Subclass.

Treatment Group:	Subclass					
	Least Symptomatic		Moderately Symptomatic		Most Symptomatic	
	FBT	SPT	FBT	SPT	FBT	SPT
	(*n =* 23)	(*n =* 12)	(*n =* 8)	(*n =* 2)	(*n =* 12)	(*n =* 2)
Highest-priority variables						
AN Criterion A Met (EDE)	0.00	0.00	1.00	1.00	1.00	1.00
AN Criterion A Met (PEDE)	0.00	0.00	1.00	1.00	1.00	1.00
AN Criterion B Met (EDE)	0.44	0.33	0.00	0.00	0.00	1.00
AN Criterion B Met (PEDE)	0.74	0.58	0.13	0.00	1.00	1.00
AN Criterion C Met (EDE)	0.96	0.75	0.50	0.00	0.58	1.00
AN Criterion C Met (PEDE)	0.96	1.00	0.63	0.50	1.00	1.00
EDE Global Score	2.58	1.98	0.36	0.27	0.89	4.13
High-priority variables						
EDE Feeling Fat	0.41	0.33	0.00	0.00	0.00	0.50
EDE Importance of Weight	0.67	0.50	0.38	0.00	0.08	1.00
EDE Importance of Shape	0.76	0.58	0.00	0.00	0.25	1.00
EDE Denial of Seriousness of Low Weight	0.68	0.45	0.25	0.00	0.36	0.00
PEDE Feeling Fat	0.36	0.30	0.00	0.00	0.11	0.00
PEDE Importance of Weight	0.91	0.75	0.29	0.50	0.67	1.00
PEDE Importance of Shape	0.91	0.75	0.14	0.00	0.89	1.00
PEDE Denial of Seriousness of Low Weight	0.77	0.75	0.63	0.00	0.75	0.50
AN Criterion D Met (EDE)	0.17	0.17	0.75	1.00	0.83	0.00
BN Criterion D Met (PEDE)	0.15	0.20	0.86	0.50	0.83	0.00
Number of Diagnostic Criteria Met (EDE)	1.52	1.17	2.13	1.00	2.25	3.00
Number of Diagnostic Criteria Met (PEDE)	1.39	1.50	2.38	1.50	2.92	3.00
PEDE Global Score	3.41	2.63	1.06	1.08	1.95	3.54
EDEQ Global Score	1.00	0.68	0.30	1.28	0.96	1.47

Table values represent means (mean scores of continues variables; means of 0/1 binary values for categorical variables, where 0 = not met and 1 = met). FBT, family-based treatment; SPT, supportive psychotherapy; Criterion A, refusal to maintain normal body weight; Criterion B, fear of weight gain; Criterion C, feeling fat and/or overvaluation of weight and/or overvaluation of shape and/or denial of seriousness of low weight; Criterion D, amenorrhea; EDE, Eating Disorder Examination; PEDE, Eating Disorder Examination**—**Parent Version; EDEQ, Eating Disorder Examination Questionnaire.

In larger non-randomized studies, subclasses can be created by estimating propensity scores (64) or *via* algorithms [e.g., (65)]. However, these methods of subclassification must be informed by clinical expertise and are only useful if the resulting subclasses contain treatment and control participants who are very similar on baseline characteristics. In a small study such as this one, using an algorithm that attempts to create balance on a large number of baseline characteristics can result in discarding most of the participants or failing to establish balance on the baseline characteristics that are most important clinically (66). Also, the resulting subclasses may not be easily interpretable.

To create baseline balance more effectively, our final design defines subclasses of participants based only on baseline AN criteria. For the purpose of developing subclasses, we used the higher symptom rating between EDE and PEDE to indicate the presence of each psychological criterion. The justification for adding the parent information (PEDE) in the generation of these subclasses is that there is both expert consensus (19) and data (67, 68) to support the idea that patient self-report, particularly for youth, is insufficient in painting the full picture of eating disorders. As getting at the “true” clinical picture for highest priority baseline characteristics is essential to create generalizable subclasses that can meaningfully combine participants from the randomized and non-randomized arms, we could not risk the marked influence of between-subject variability in denial and minimization on self-report measurement. For similar reasons, we ignored amenorrhea because it is a controversial diagnostic criterion without consistent diagnostic validity that was ultimately eliminated in DSM-5 during the course of this study (69).

There were three subclasses generated from this process. The first was the least symptomatic in terms of AN criteria, in that criteria B and/or C were met but not A (see **Table 1** for a review of the criteria). The second was a moderately symptomatic subclass, who met criterion A alone or in combination with B or C. The third was the most symptomatic subclass, who met DSM-IV AN criteria A, B, and C at baseline.

We then checked that, within each of the three subclasses we created, FBT and SPT participants were similar not only on baseline AN criteria but also on the other baseline characteristics that are clinically relevant. Thus, while subclasses were based on diagnostic profile, balance was checked on all of the baseline characteristics. Balance was perfect or very good on the highest priority characteristics, at the expense of relatively larger baseline differences on characteristics that were initially categorized as lower priority. Given the very small sample sizes, this tradeoff between creating balance on certain characteristics versus other characteristics was expected. A benefit of the design we chose is that each of the subclasses reflects a well-defined segment of SAN (see *Results*), allowing us to estimate the effects of FBT versus SPT within these sub-samples. Our strategy of subclassifying on key baseline criteria is in many ways similar to coarsened exact matching (70).

#### Effects Estimates

For each outcome, by subclass, we report raw end-of-treatment effect size, calculated as the difference between end-of-treatment means in the FBT and SPT groups within a subclass, divided by the baseline standard deviation of the same variable for all 59 participants.

The statistical analysis relied on longitudinal mixed effects models, following the original analysis plan, with subclass as a key predictor, reflecting the partially-randomized preference design and creation of subclasses to establish covariate balance. All participants were included in analyses, including those who dropped out or were exited from the study. The final analyses focus on weight gain and psychological outcomes, as planned, but not conversion to AN, even though conversion to AN was a planned primary outcome. Only two participants converted to AN during the study (one prior to session 1 and one at session 3), and so it is not possible to draw meaningful conclusions about the effects of the two treatments on conversion to AN.

We fit longitudinal mixed effects linear regression models to the continuous primary outcome variables (percent EBW and EDE Fear of Weight Gain, Feeling Fat, Importance of Weight, and Importance of Shape using MIXREG software) (71). The mixed effects models implemented by Hedeker and Gibbons are specifically intended for psychiatric data and have several characteristics that provide solutions to commonly observed problems in the analysis of longitudinal data, including missing data and serial correlation (72). In addition, these analyses can model systematic person-specific deviations from the average time trend.

Specifically, we used random effects regression to model each outcome as a function of treatment, time, treatment x time, subclass, subclass x treatment, and subclass x time. We modeled the individual response of each participant as a line with intercept (baseline response) and slope (improvement rate), obtaining an average trend line for each intervention group, by subclass. We report contrasts from the mixed effect models estimating the effects of FBT versus SPT by the end of treatment. We do not report *p*-values, because this pilot study was not powered to show significant effects, particularly given that subclassification was needed to establish baseline balance. Had we calculated *p*-values or corresponding confidence intervals, they would have been large, primarily driven by the small sample size rather than the effect sizes. Following recent statements from the field of statistics (73–75), we focus on effect estimates to generate hypotheses that can be tested in future, larger studies.

## Results

**Figure 1:**
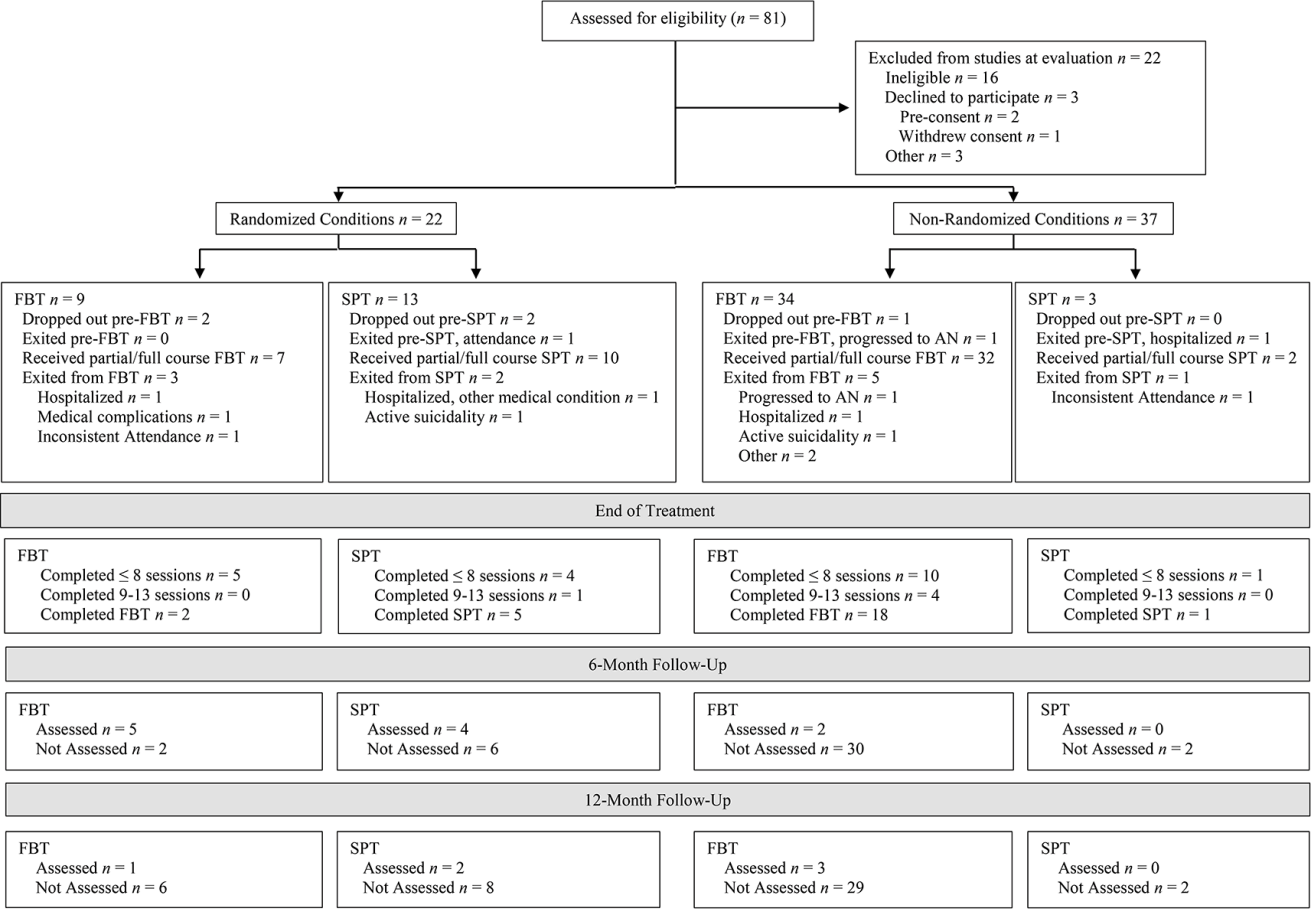
Participant flow chart following Consolidated Standards of Reporting Trials guidelines for family-based treatment (FBT) and supportive psychotherapy (SPT) conditions in the randomized and non-randomized study arms.

Fifty-nine participants were entered into the primary or parallel study (see **Figure 1**). The most frequent source of referral was pediatricians (*n* = 23, 39.0%). **Table 3** shows the baseline demographic and clinical characteristics of the overall sample as well as by treatment group and randomization status. Rates of randomization (versus non-randomization study entry) were similar across the three subclasses depicted in **Table 2**: 40% (14/35) for the least symptomatic group, 30% (3/10) for the moderately symptomatic group, and 36% (5/14) for the most symptomatic group. The research was conducted from September 2005 to August 2011.

**Table 3:** Demographic and clinical characteristics at baseline, overall, and by treatment group and randomization status.

	FBT		SPT		Total
	Randomized (*n* = 9)	Non-Randomized (*n* = 34)	Randomized (*n* = 13)	Non-Randomized (*n* = 3)	Overall Sample (*n* = 59)
Demographics					
Age (years)	14.01 (1.96)	13.13 (2.03)	14.66 (2.17)	15.16 (0.56)	13.70 (2.09)
Self-Identified Gender: Female	9.00(100.00)	28 (82.40)	10.00 (76.90)	3.00 (100.00)	50.00 (84.70)
Self-Identified Race/Ethnicity					
White	7.00 (77.80)	29.00 (85.30)	11.00 (84.60)	3 (100.00)	50.00 (84.70)
Hispanic/Latino	2.00 (22.20)	2.00 (5.90)	2.00 (15.40)	0.00 (0.00)	6.00 (10.20)
Asian	0.00 (0.00)	2.00 (5.90)	0.00 (0.00)	0.00 (0.00)	2.00 (3.40)
Clinical Characteristics					
Weight (pounds)	94.84 (24.21)	86.11 (20.42)	101.25 (23.70)	109.67 (2.70)	91.97 (22.17)
Height (inches)	61.97 (4.60)	60.78 (4.88)	62.97 (3.97)	64.83 (1.61)	61.65 (4.60)
%EBW	88.33 (9.16)	86.22 (8.53)	90.67 (12.81)	91.94 (2.42)	87.82 (9.56)
Duration of Symptoms (months)	15.89 (9.48)	14.18 (16.10)	10.08 (8.97)	18.34 (3.51)	13.74 (14.02)
Current Treatment (frequency)	3.00 (33.33)	16.00 (47.10)	4.00 (30.80)	1.00 (33.33)	24.00 (40.70)
Prior Hospitalizations (total count)	0.25 (0.46)	0.41 (0.99)	0.15 (0.55)	0.33 (0.58)	0.33 (0.82)
Courses of Treatment (total count)	1.22 (1.20)	1.21 (1.19)	0.77 (0.73)	1.67 (0.57)	1.14 (1.08)
EDE Restraint	1.84 (1.69)	1.84 (1.99)	1.70 (2.29)	3.07 (2.65)	1.88 (2.01)
EDE Eating Concern	1.11 (1.21)	0.98 (1.24)	1.32 (1.52)	2.40 (2.09)	1.15 (1.35)
EDE Weight Concern	1.77 (2.01)	1.72 (1.86)	1.96 (1.85)	3.73 (1.50)	1.88 (1.87)
EDE Shape Concern	1.79 (1.92)	2.19 (2.12)	2.03 (2.09)	3.75 (2.00)	2.17 (2.06)
EDE Global Score	1.63 (1.60)	1.77 (1.70)	1.75 (1.82)	3.24 (2.01)	1.82 (1.71)
PEDE Restraint	3.04 (0.97)	3.04 (1.74)	3.16 (1.66)	4.10 (0.36)	3.12 (1.57)
PEDE Eating Concern	1.41 (1.55)	1.46 (1.33)	1.24 (1.63)	1.47 (0.83)	1.40 (1.39)
PEDE Weight Concern	1.92 (1.49)	2.13 (1.86)	2.94 (2.02)	3.67 (2.04)	2.35 (1.87)
PEDE Shape Concern	2.38 (1.58)	2.74 (2.04)	2.52 (2.12)	2.84 (2.00)	2.64 (1.95)
PEDE Global Score	2.19 (1.07)	2.67 (2.38)	2.45 (1.60)	3.02 (0.36)	2.56 (1.99)
EDEQ Restraint	2.96 (1.50)	2.04 (1.88)	2.03 (1.80)	3.07 (1.81)	2.24 (1.80)
EDEQ Eating Concern	2.01 (1.65)	1.28 (1.43)	1.33 (1.33)	3.00 (2.25)	1.51 (1.51)
EDEQ Weight Concern	2.62 (1.79)	2.10 (1.80)	2.40 (2.09)	3.73 (1.40)	2.34 (1.84)
EDEQ Shape Concern	2.69 (1.74)	2.41 (1.99)	2.19 (2.16)	4.10 (1.58)	2.49 (1.97)
EDEQ Global Score	2.57 (1.55)	1.96 (1.66)	1.93 (1.75)	3.47 (1.61)	2.13 (1.67)

Table values represent mean (standard deviation) for continuous variables, and frequency (percent) for categorical variables. FBT, family-based treatment; SPT, supportive psychotherapy; %EBW, percent expected body weight (weight corresponding to the 50^th^ percentile body mass index for age and sex); EDE, Eating Disorder Examination; PEDE, Eating Disorder Examination**—**Parent Version; EDEQ, Eating Disorder Examination Questionnaire.

For each outcome, by subclass, **Table 4** shows baseline means, the raw difference between FBT and SPT end-of-treatment means, the effect size calculated by standardizing the raw end-of-treatment difference by the baseline standard deviation, and contrasts from the mixed effects model comparing FBT and SPT. Ranges (minimum and maximum values) for baseline and end-of-treatment means are reported as measures of variability instead of standard deviations, which would not indicate variability well for the subgroups with an *n* of 2.

**Table 4:** Study Results.

Treatment group	Subclass								
	Least Symptomatic			Moderately Symptomatic			Most Symptomatic		
	FBT	SPT	FBT—SPT	FBT	SPT	FBT—SPT	FBT	SPT	FBT—SPT
	(*n* = 23)	(*n =* 12)	Difference	(*n =* 8)	(*n* = 2)	Difference	(*n* = 12)	(*n* = 2)	Difference
Percent EBW									
Baseline									
Mean	92.29	94.69		77.27	79.55		82.13	79.76	
Minimum	85.55	86.05		71.42	74.10		76.20	78.31	
Maximum	109.31	121.43		84.78	85.00		88.93	81.22	
EOT									
Mean	96.81	101.07	-4.26	84.52	77.88	6.65	88.02	86.10	1.92
Minimum	83.70	86.07		71.39	74.10		78.07	85.32	
Maximum	113.74	126.42		93.20	81.65		101.44	86.89	
Effect Size^			-0.45			0.70			0.20
Contrast at EOT^*^			-3.47			0.02^#^			2.95
EDE Fear of Weight Gain									
Baseline									
Mean	3.48	2.17		0.25	0.00		0.75	6.00	
Minimum	0.00	0.00		0.00	0.00		0.00	6.00	
Maximum	6.00	6.00		2.00	0.00		4.00	6.00	
EOT									
Mean	3.26	2.67	0.59	0.38	0.00	0.38	0.83	3.00	-2.17
Minimum	0.00	0.00		0.00	0.00		0.00	0.00	
Maximum	6.00	6.00		3.00	0.00		6.00	6.00	
Effect Size^			0.23			0.14			-0.83
Contrast at EOT*			1.04			0.29			-4.14
EDE Feeling Fat									
Baseline									
Mean	2.96	1.83		0.25	0.00		0.92	3.50	
Minimum	0.00	0.00		0.00	0.00		0.00	1.00	
Maximum	6.00	6.00		2.00	0.00		5.00	6.00	
EOT									
Mean	2.74	2.50	0.24	0.38	0.00	0.38	0.67	3.00	-2.33
Minimum	0.00	0.00		0.00	0.00		0.00	0.00	
Maximum	6.00	6.00		3.00	0.00		6.00	6.00	
Effect Size^			0.10			0.15			-0.95
Contrast at EOT*			0.52			-0.23			-3.05
EDE Importance of Weight									
Baseline									
Mean	4.04	3.67		2.38	2.00		1.75	5.50	
Minimum	0.00	1.00		0.00	1.00		0.00	5.00	
Maximum	6.00	6.00		5.00	3.00		6.00	6.00	
EOT									
Mean	3.17	2.75	0.42	0.38	1.50	-1.12	0.83	3.50	-2.67
Minimum	0.00	0.00		0.00	0.00		0.00	1.00	
Maximum	6.00	6.00		1.00	3.00		6.00	6.00	
Effect Size^			0.20			-0.54			-1.27
Contrast at EOT^*^			0.35			-0.06			-3.32
EDE Importance of Shape									
Baseline									
Mean	4.35	4.00		1.25	1.00		2.17	6.00	
Minimum	0.00	1.00		0.00	0.00		0.00	6.00	
Maximum	6.00	6.00		4.00	2.00		5.00	6.00	
EOT									
Mean	3.48	2.83	0.64	0.62	0.00	0.62	0.83	4.00	-3.17
Minimum	0.00	0.00		0.00	0.00		0.00	2.00	
Maximum	6.00	6.00		2.00	0.00		5.00	6.00	
Effect Size^			0.29			0.28			-1.44
Contrast at EOT^*^			0.65			0.73			-3.28

FBT, family-based treatment; SPT, supportive psychotherapy; EBW, expected body weight; EOT, end-of-treatment; EDE, Eating Disorder Examination.

^Effect sizes are end-of-treatment differences in means, divided by standard deviation of all 59 participants at baseline.

*Contrasts are estimates of FBT v. SPT effects by the end of treatment, by subclass, according to a longitudinal mixed effect model.

^#^This contrast is calculated at session 4 rather than end of treatment because of the very early attrition of the two participants in the A+B/C SPT group.

**Figure 2:**
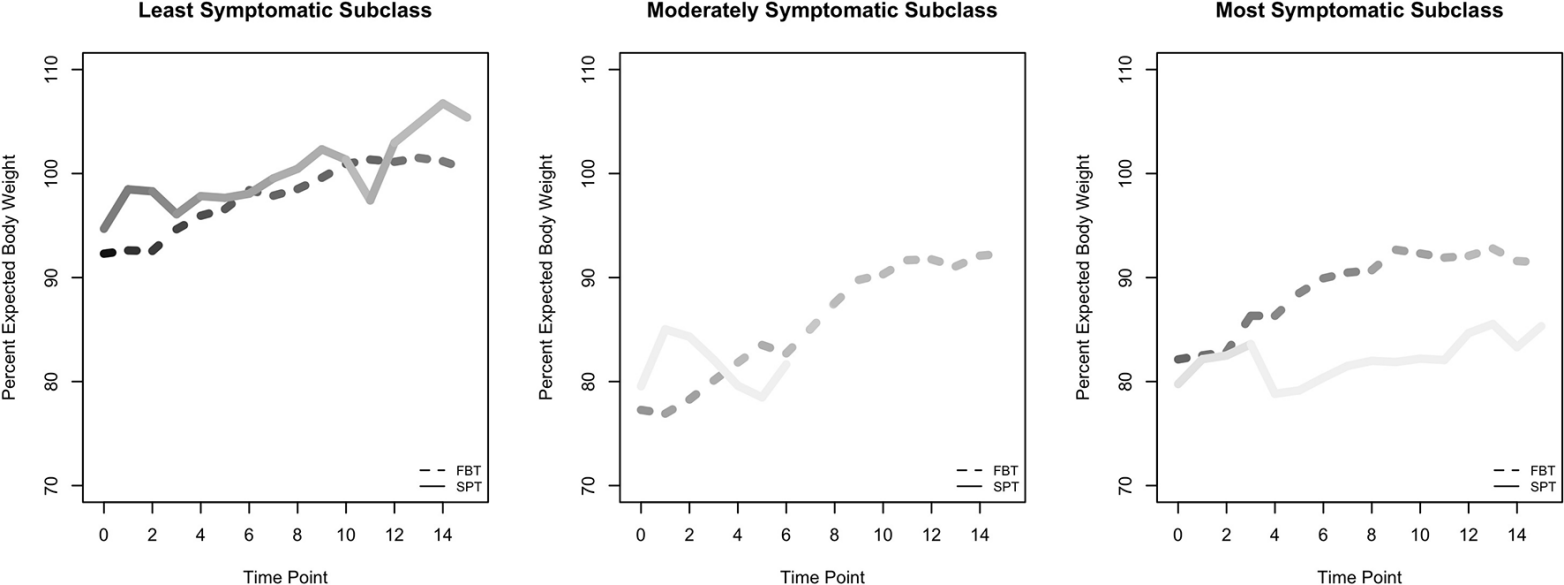
Raw means for percent expected body weight (EBW) outcome, by subclass. Figures of time series for psychological outcomes are not shown because psychological outcomes were measured at only two time points. Lines are shaded to reflect the number of participants in each group, and at each time point within each group, with darker shading indicating greater numbers.

There were substantially more participants in the least symptomatic group, in which B and/or C were met but not A, than in the other two subclasses (**Table 2**). Among these participants, individuals who received SPT exhibited higher percent EBW over time than those who received FBT (**Table 4** and **Figure 2**). The model-estimated effects on psychological outcomes were relatively small, with individuals in SPT possibly experiencing greater reductions than those in FBT.

For participants in the moderate subclass, who met criterion A alone or in combination with B or C, the model-estimated effect after four sessions suggests that participants in the two treatments were similar on percent EBW and on psychological outcomes (**Table 4** and **Figure 2**). We calculated an early-session (4) contrast here rather than an end-of-treatment contrast for percent EBW because of the very rapid attrition of the two SPT participants in the subclass.

For participants in the most symptomatic subclass, who met DSM-IV AN criteria A, B, and C at baseline, the model results show that by the end of treatment, individuals who received FBT had a higher percent EBW than those who received SPT (**Figure 2**). In addition, individuals in this subclass who received FBT ended treatment with a greater reduction in psychological symptoms (EDE Fear of Weight Gain, Feeling Fat, Importance of Weight, and Importance of Shape) than individuals in SPT. We note that the SPT and FBT participants were imperfectly balanced at baseline on these continuous psychological measures, even though they all met the categorical psychological criteria at baseline. This discrepancy is a function of criterion C's compound algorithm capturing disturbance in experience of body shape or weight, and/or undue influence of shape or weight on self-concept, and/or denial of seriousness of illness.

## Discussion

Early intervention is considered to confer an improved prognosis among children and adolescents exhibiting signs and symptoms of AN (11–15). This study adapted and explored FBT for application at the intersection of prevention and treatment, for youth with potentially prodromal AN. The pattern of symptom observations over time was dependent on subclass of SAN per DSM-IV criteria and on target outcome variable category (weight or psychological). Among those participants in the most symptomatic group, who would meet full AN diagnosis according to the more developmentally sensitive DSM-5 criteria, FBT participants experienced greater reversal of weight loss than SPT participants. For the least symptomatic group, SPT participants experienced greater weight loss correction than FBT participants. In the moderate group, weight outcomes were similar in FBT and SPT. For psychological outcomes, no clear or strong differences between FBT and SPT were evident.

These observations within the SAN population are consistent with a robust treatment literature demonstrating the efficacy of FBT in achieving weight restoration for youth with diagnostic-level AN (35, 76). Findings also align with research highlighting that specialty treatments for AN, relative to comparator interventions, more consistently achieve improvements for weight-based AN symptoms than for psychological features of the illness (77). Results should be considered in the context of the pilot nature of the study, its primary goals to observe effect estimates and feasibility indicators, and the small sample sizes. In particular, the SPT groups in the moderate and severe subclasses each had an *n* of 2, challenging confidence in their findings. Combining subclasses to increase treatment group sizes for analysis was not possible, as doing so would have imbalanced FBT and SPT participants on baseline covariates. Balance between subclasses is a prerequisite to inferring causality, and violating balance would pose an even greater problem than small group size. However, this pilot study aimed to inform the design and hypotheses of future, larger research trials (34) on early treatment strategies, rather than to draw definitive conclusions about treatment efficacy.

Conversion from SAN to AN during the observation period was rare (**Figure 1**), although attrition compromises interpretability of this finding. That said, the two cases who did progress to the full diagnosis worsened quickly after presentation to the study, one before the first treatment session and the other by the third session. Similarly, participants who exited the study because they deteriorated and required hospitalization for their eating disorder symptoms**—**while not converting to AN—did so early (one before the first session and two by the third). Another three participants were exited for other reasons of clinical significance (one for medical instability that did not require hospitalization, and two for active suicidality). Thus, a total of 8/59 (13.6%) participants experienced marked clinical decline, even under close observation and care. This pattern, which spanned the least to most symptomatic subclasses, underscores the importance of early intervention among children and adolescents exhibiting proximal risk by virtue of their clinically significant, diagnostic AN symptoms. This also validates the clinical concerns identified by pediatricians, who constituted the most common referral source for the study.

The observed clinical deterioration, and the fact that it was not exclusive to the most sick subclass, speaks to the composition of the study sample. Inclusion criteria, while deliberately broad in certain respects, were also designed to capture a specific cross-section of children and adolescents already exhibiting core clinically significant symptoms of AN rather than only risk factors. They were also treatment-seeking or treatment-referred, in contrast to the sample in the Jacobi et al. (32) community parent-based, internet-delivered prevention study. Perhaps not surprisingly then, participants in the current study had already had some exposure to eating disorders treatment—approximately once, a rate that included treatment before or at the time of presentation, and that was fairly consistent across randomized and non-randomized study arms, treatment groups, and subclasses. Interestingly, Jacobi and colleagues (32) encountered significant recruitment, engagement, and retention challenges; when parents declined participation after being informed that their child had screened positively for AN risk, the majority cited lack of concern about the identified risk factors (including weight loss) and therefore no interest in a prevention program, and many stated that their physicians had no concerns about their child's risk or had even advised the parents against participating. This suggests that AN researchers should consider recruitment feasibility in addition to clinical impact when identifying proximal risk definitions and early intervention timing and methods. In addition, future research on youth at risk for AN should take into account the likely heterogeneity of the study sample and anticipate a restricted range of potential improvement in particular target outcome variables for some cases (e.g., less weight gain in those with less baseline weight loss). Broader outcome indices, such as quality of life, that may be more uniformly applicable could be included.

Beyond the study's implications for further research on early identification and treatment strategies for AN-spectrum presentations, the innovative partially randomized preference design has compelling application to broader AN intervention research, which is known for its recruitment challenges (78, 79). Adults may decline to be randomized because of fear that one treatment may yield greater weight gain than another, an outcome about which individuals with AN are ambivalent. Conversely, parents of children and adolescents might decline consent for randomization for their children because of motivation to receive a specific intervention, as was the case in the current study and which represents a critical feasibility finding. Specifically, as described earlier, during the early stages of the research, a clear and rising preference for FBT among carers of potential participants emerged, resulting in a high rate of declining randomization. With the addition of the parallel arm of the study, approximately two-thirds (37/59) of the ultimate sample were non-randomized participants. Of these, over 90% (34/37) elected to receive FBT. The partially randomized preference design is a strength of the study in that it has the benefit of retaining data from participants who would otherwise remain unobserved. In addition, the unbiased process of creating balanced subclasses of participants permits an empirically-derived convergence of data from the two parallel study arms for final analysis. AN research could also benefit from other innovative designs for evaluative complex interventions, beyond the partially randomized preference model (80).

The small sample size, especially given the lack of randomization for many participants, limited both the power of the study and the ability to create subclasses that were perfectly balanced on all baseline characteristics. Our choice to prioritize balance on the A, B, and C baseline criteria necessarily resulted in less balance on other baseline variables. A larger sample size within a partially randomized preference trial design would allow for better baseline balance, and *p*-value or confidence interval calculations in addition to effect sizes. While we obtained good balance between treatment groups on the highest- and high-priority covariates in the subclass generation, greater homogeneity could be achieved with a larger sample size. It would also permit separate analyses of randomized and non-randomized groups, beyond treatment comparisons within and across the subclass formations.

Attrition is an additional feasibility finding and study limitation, especially its rate during follow-up, which prevented data analysis beyond the active intervention observation period. However, we do not know whether for certain cases, attrition was a function of limited need for treatment (i.e., by virtue of rapid response or transience of symptoms) as opposed to lack of engagement. Future research evaluating this could approach the question from both clinical and healthcare cost perspectives, and include mixed methods to gather qualitative data on parents' and patients' experiences in addition to quantitative data. Notably, rates of treatment completion among those participants who were not exited by the study team for clinical deterioration**—**a proxy for engagement**—**were similar between FBT and SPT, at approximately 50%. Importantly, while the present study design aimed to control for non-specific therapy effects in the form of SPT, it did not control purely for the effect of time with a wait-list control condition. Thus, the course of illness of SAN in the absence of any intervention**—**in itself and as compared to specialty clinical attention—cannot be inferred from this research. In addition, this study cannot elucidate symptom course among non-treatment-seeking samples.

One aspect of the hybrid nature of the current study**—**in that it incorporated elements of both efficacy and effectiveness trials**—**was the decision to permit participants to remain in outside treatment, e.g., psychopharmacological intervention. This allowance functions as a limitation while also increasing the pilot study's ecological validity. Another limitation of the study was that we did not collect data on the treatment preferences, if any, of the participants who agreed to be randomized. Thus, we only know the preferences of those in the non-randomized arm, and we could not analyze overall whether being matched to one's preferred treatment confers a better prognosis. The only related data we have is that there was a similar percentage of participants declining randomization in each subclass, although we did not attempt to balance the subclasses on this variable. Several further study limitations were secondary to resource limitations in the context of the grant mechanism and pilot status of the trial. These include outcome assessments being conducted by research assistants who were at times aware of randomization status or treatment arm, and the lack of inter-rater reliability data for assessment measures.

Considering feasibility findings, observations of clinical deterioration across the sample, and effect estimates from the current study all together, future trial designs with the SAN population would (a) hypothesize that utility of family-level interventions, particularly for more symptomatic patients, and with justification for close clinical monitoring and attention and (b) anticipate families' reluctance to be randomized to a less active treatment like SPT.

Future research investigating children and adolescents with potentially prodromal AN should also adjust the definition of SAN in accordance with DSM-5 and further-evolving diagnostic criteria that may better delineate between true “caseness” in youth versus sub-syndromal, high risk presentations, including those that meet criteria for a DSM-5 Other Specified Feeding or Eating Disorder (OSFED). In particular, understanding how atypical AN, one such clinically significant subcategory of OSFED that has appropriately garnered significant recent attention, fits into the paradigm of risk, prevention, and course of AN will also be critical. Further planned analyses with data from the current study seek to investigate SAN through the dual prisms of changes in diagnostic systems (DSM-IV to DSM-5) and symptom informants (patients versus parents). In addition, while controlling for the effects of time alone, without clinical attention, in SAN research is advantageous for causal inference of results, ethical considerations challenge this design. This may be particularly true for treatment-seeking and treatment-referred samples given their proximal risk of clinical deterioration or conversion to AN.

## Data Availability Statement

The datasets generated for this study are available on request to the corresponding author.

## Ethics Statement

The research involving human participants was reviewed and approved by Icahn School of Medicine at Mount Sinai. Written informed consent for children to participate in this study was provided by the participants' parents/legal guardians.

## Author Contributions

KL, RW, SM, CP, NZ, DG, JL, JN, CT, and BW all provided substantial contributions to the conception and design of the study. KL, SM, CP, LH, KK, and DS contributed to data analysis. KL, LH, and BW contributed to data acquisition processes. All authors contributed to interpretation of data and to drafting and/or critically reviewing/revising the paper for important intellectual content. All authors approved the submitted version of the manuscript.

## Funding

This research was supported by a grant from the National Institute of Mental Health, K23 MH074506 (PI: KL; ClinicalTrials.gov NCT00418977, Early Identification and Treatment of Anorexia Nervosa^2^), with Jack Gorman and Dennis Charney serving sequentially as institutional sponsors of the award at the Icahn School of Medicine at Mount Sinai.

## Conflict of Interest

KL reports receiving royalties from Routledge, and is a faculty member of and consultant for the Training Institute for Child and Adolescent Eating Disorders. RW reports receiving stipend from Wiley & Sons as editor of the International Journal of Eating Disorders. NZ and DG reports receiving royalties from Guilford Press and Routledge, and is co-director of the Training Institute for Child and Adolescent Eating Disorders, LLC. JL reports receiving royalties from Guilford Press and Routledge, and is co-director of the Training Institute for Child and Adolescent Eating Disorders, LLC. JN reports the following disclosures within the past year: advisor and/or consultant for Adlon Therapeutics, Akili Interactive, Arbor, Medice, NLS Pharmaceutics, Rhodes, Takeda, and Supernus; DSMB member for Pfizer and Sunovion; research support from Otsuka, Shire and Supernus; speaker fees from Takeda for disease-state presentations. CT and BW report receiving royalties and honoraria from Guilford Press, McGraw-Hill, Oxford University Press, UpToDate, British Medical Journal, Johns Hopkins Press, and Guidepoint Global.

The remaining authors declare that the research was conducted in the absence of any commercial or financial relationships that could be construed as a potential conflict of interest.

## Footnotes

1 Summary table of moderate- and low-priority variables, as well as data on final balance achieved on all variables, are available upon request.

2 See Study Record Versions 1-8 for accurate identification of primary outcome measures; Versions 9 and 10 contain locked clerical errors.
